# Multilevel animal societies can emerge from cultural transmission

**DOI:** 10.1038/ncomms9091

**Published:** 2015-09-08

**Authors:** Maurício Cantor, Lauren G. Shoemaker, Reniel B. Cabral, César O. Flores, Melinda Varga, Hal Whitehead

**Affiliations:** 1 Department of Biology, Dalhousie University, 1355 Oxford Street, Halifax, Canada, NS B3H 4J1; 2 Department of Ecology and Evolutionary Biology, University of Colorado at Boulder, UCB 334, Ramaley Hall, Boulder, Colorado 80309, USA; 3 National Institute of Physics, College of Science, University of the Philippines Diliman, Quezon City 1101, Philippines; 4 Sustainable Fisheries Group, Bren School of Environmental Science and Management and Marine Science Institute, University of California Santa Barbara, 2400 Bren Hall, California 93106, USA; 5 School of Physics, Georgia Institute of Technology, 837 State Street, Atlanta, Georgia 30332-430, USA; 6 Department of Physics, University of Notre Dame, 225 Nieuwland Science Hall, Notre Dame, Indiana 46556, USA

## Abstract

Multilevel societies, containing hierarchically nested social levels, are remarkable social structures whose origins are unclear. The social relationships of sperm whales are organized in a multilevel society with an upper level composed of clans of individuals communicating using similar patterns of clicks (codas). Using agent-based models informed by an 18-year empirical study, we show that clans are unlikely products of stochastic processes (genetic or cultural drift) but likely originate from cultural transmission via biased social learning of codas. Distinct clusters of individuals with similar acoustic repertoires, mirroring the empirical clans, emerge when whales learn preferentially the most common codas (conformism) from behaviourally similar individuals (homophily). Cultural transmission seems key in the partitioning of sperm whales into sympatric clans. These findings suggest that processes similar to those that generate complex human cultures could not only be at play in non-human societies but also create multilevel social structures in the wild.

## Abstract (web summary)

Multilevel societies are comprised of hierarchically nested levels of social organization, but how they arise is not well understood. Here Cantor *et al*. find that the emergence of sperm whale clans is more likely to be driven by cultural transmission of acoustic repertoires than via stochastic processes.

## Graphical abstract

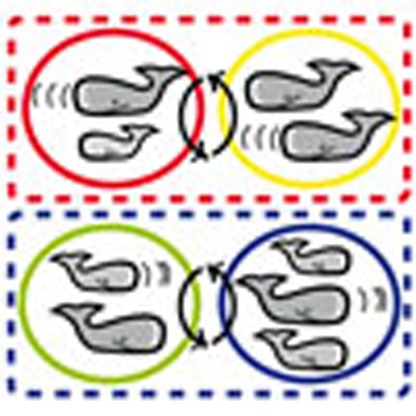

Selection for effective reproduction, foraging and survival shapes social interactions and relationships—both in current ecological conditions and historic evolutionary pathways—and produces a variety of animal social structures^1^,^2^,^3^. Multilevel societies, consisting of hierarchically nested social levels, are particularly interesting as they suggest multilevel selection. Such societies are found in human^4^,^5^ and non-human primates^6^,^7^, African elephants^7^,^8^ and orca whales^7^,^9^. The origins of multilevel social structures remain unclear, and are likely to be complex^4^,^5^,^6^,^7^. They likely include general drivers of social patterns (the ecological, evolutionary and social contexts regulating group-living trade-offs^10^,^11^) as well as individual variation in behaviour^3^ and the time and cognitive constraints involved in managing multiple social relationships^12^,^13^.

A frequently neglected potential driver of animal sociality is culture—socially learned behaviour shared within subsets of a population^14^. Experiments in captivity and the wild, as well as long-term observational studies and computer simulations, all suggest that culture can be important drivers of phenotypic variation in several animal taxa^14^,^15^ and shape social structures^16^. One hypothesized effect occurs when individuals who behave similarly preferentially interact (homophily)^17^ and thus learn from each other, resulting in groups or sub-populations with increasingly homogenous behaviour^18^. Particularly strong homogenization may occur when individuals disproportionately learn the most common behavioural variant from their social contacts (conformism) to assist group integration or because a well-spread behavioural trait may generally be adaptive^19^. Since social relationships are cognitively and energetically costly, particular cultural behaviours can be used to mark the identity of a social group (symbolic marking)^20^,^21^, which facilitate interactions among individuals who behave similarly. Therefore, biases such as conformism, homophily and symbolic marking affect the learning of behaviours among individuals^22^,^23^ segregating them into groups with increasingly distinctive behavioural patterns^16^,^24^,^25^,^26^. Examples include the cultural boundaries delineated by specialized foraging techniques and traditions^27^,^28^, and distinct dialects and communication signals^29^,^30^ observed in primates, birds and cetaceans.

Cultural segregation can naturally follow geographic segregation^31^,^32^. With little or no contact between sets of individuals, behavioural repertoires tend to diverge over time. Therefore, pure spatial and/or demographic factors can lead to the accumulation of behavioural variations, with drift over time generating behavioural heterogeneity between sets of individuals^33^. Typically, the more distant these sets of individuals are^31^, and the lower the levels of dispersal, migration and population mixing among them^34^, the more divergent their cultural traits^32^,^35^. Among allopatric cultural groups, large distances or limited movement of individuals can complicate the understanding of true drivers of behavioural divergence, since environmental and genetic differences make it difficult to isolate cultural determinants of behavioural variation^15^. We know little about how the behaviourally distinct animal groups evolve and are maintained in sympatry, but in a common environment the effects of culture can be clearer (for human examples, see refs 23, 26, 36).

Long-term observational studies have unveiled social complexity^37^ and cultural diversity among cetaceans using the same waters at the same time^38^. For instance, female sperm whales (*Physeter macrocephalus*) form matrilineally based social units with about 12 members each^39^. These units are organized into clans with distinctive behaviour in several realms, including vocal repertoires^30^, creating a multilevel society^39^. Social units have characteristic repertoires of codas^40^—patterns of clicks used for communication—and unit members are observed to only group with other social units from their own clan, with whom they have similar coda repertoires^30^. In sperm whales, as in other cetaceans, information flow through social learning is a key driver of behaviour^38^. As learned acoustic signals can be important in social relationship mediation^41^, they can also possibly shape social structure^16^; for instance, learned vocalizations are hypothesized to underlay the partition of sperm whales into clans^30^,^39^. The sperm whale clans in the Pacific are sympatric^30^,^42^ and include genetically similar individuals of all ages^43^, thus systematic differences between them are likely to be cultural, representing a good model for investigating how the sympatric animal cultural groups form in the wild.

Here we investigate the mechanisms giving rise to the nested social levels of sperm whale society observed empirically. We build mechanistic agent-based models, using empirical data collected over 18 years in offshore waters of the eastern Pacific Ocean, to test whether clans could emerge in sympatry solely via cultural or genetic drift of communication signals over time or whether social learning is required. We also consider more sophisticate scenarios in which social learning is biased by homophily, conformism and/or symbolic marking. With homophily, communication similarity drives social relationships, so coda learning is preferentially from individuals with similar vocal repertoires^17^. With conformism, the most common coda types that an individual is exposed to are learned disproportionately more often^18^. In symbolic marking, particular codas are used by all members of a social entity^20^. We accounted for different degrees of population mixing by allowing coda transmission processes to operate in the entire population, within social units or within pre-existent allopatric clans, such as the geographically based clans observed in the North Atlantic^39^,^44^. Our investigation of the social patterns that emerge from animal collective behaviour is the first formal effort to our knowledge to relate the formation of multilevel societies to cultural evolution. We show that the higher social level of sperm whale societies unlikely originates from stochastic processes but rather from biased cultural transmission of acoustic communication signals.

## Results

### Empirical social patterns

**Figure 1:**
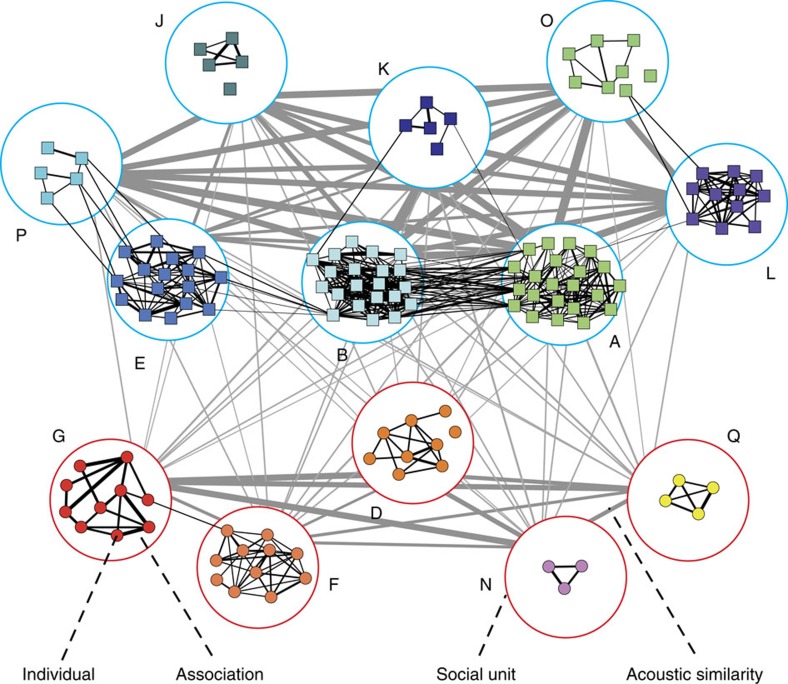
Empirical multilevel network depicting the three nested levels in the sperm whale society off the Galápagos Islands: individuals within social units within vocal clans. In the social network, modules of individual whales (coloured small nodes) connected by their social relationships (black lines with thicknesses proportional to the time individuals were identified in the same group) define the social units (letter-labelled large nodes). In the overlapped acoustic network, modules of social units connected by the similarity in acoustic behaviour (grey lines whose thicknesses are proportional to multivariate similarity of coda repertoires) represent the vocal clans (blue: Regular clan, characterized by codas with regularly-spaced clicks; red: Plus-One clan, characterized by codas with extended pause before the final click^35^). Social relationships and acoustic similarities are replotted results from refs 35, 52, respectively.

Over our 18-year study of sperm whales in the Eastern Pacific, the sperm whale society showed a hierarchical structure with three conspicuous nested levels: individuals in social units forming vocal clans (Fig. 1). The social network of photo-identified individuals (nodes) connected by their social relationships (weighted links estimated by half-weight association indices) displayed a modular topology (*Q*=0.886), in which modules of highly connected nodes delineated social units of individuals that live and move together for several years^45^. These modules representing social units formed larger modules in an overlapped acoustic network of social units (nodes) connected by acoustic behaviour similarity (weighted links estimated by multivariate similarity of coda repertoires). The acoustic network also displayed a modular topology (*Q*=0.154, 95% confidence interval=0–0.124): here modules of social units with shared coda repertoires depicted the vocal clans^30^. The multilevel sperm whale society exhibited high within-clan acoustic similarity but very low between-clan acoustic similarity, and no between-clan social interactions (Fig. 1).

### Emergent social patterns in simulations

**Figure 2:**
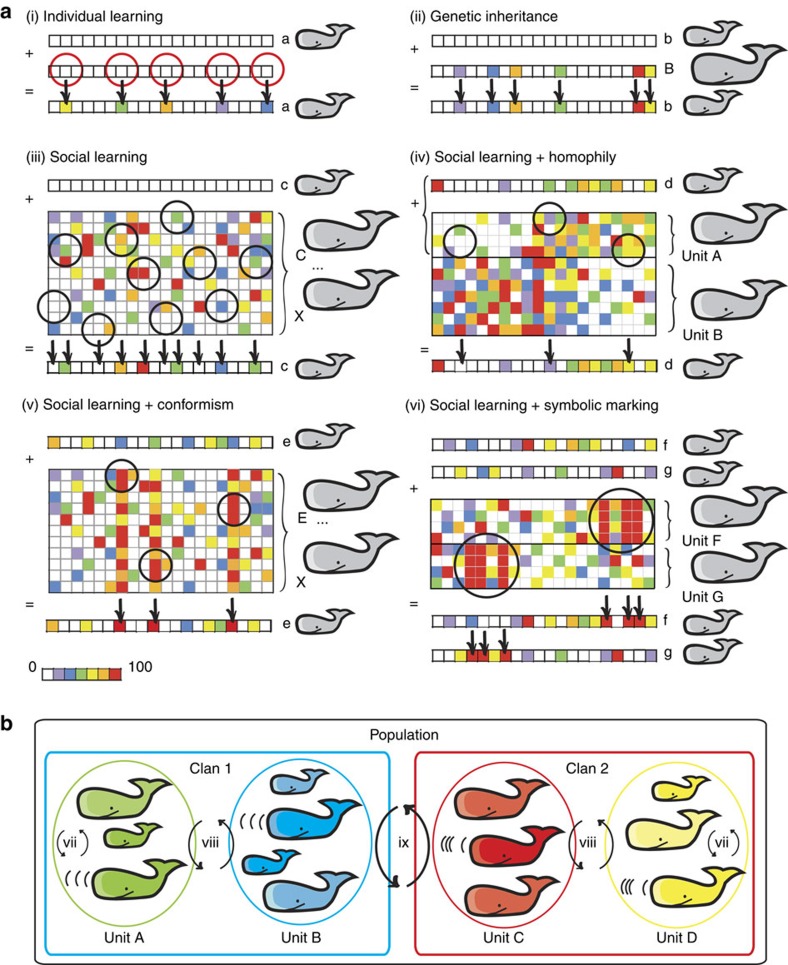
Schematic representation of the agent-based models. (**a**) Coda transmission mechanisms are represented as changes in the coda repertoires vector (squares: coda types; colours: frequency of usage: absent=0, always=100%). Calf agents change repertoires three times (between 0 and 2 year old) under one of following mechanisms. (i) Individual learning: newborn agent *a* starts with an empty coda vector; half of the elements are randomly selected to receive absolute frequencies of usage from a uniform distribution ∈[0,100]. (ii) Genetic inheritance: newborn agent *b* starts with an empty coda vector, which is filled with the same coda types and frequencies of its mother *B*. (iii) Oblique social learning: newborn agent *c* starts with an empty coda vector; at the age 0 year it randomly samples 62 elements (including zeroed elements) from the coda vector of other adult agents, kin-related or not; at ages 1 and 2 years, the calf repeats the process, replacing a portion of sampled elements. For iv–vi, calves gain an initial repertoire via oblique social learning, then at ages 1 and 2 years, the following effects were included. (iv) Homophily: calf *d* copies from adult agents of the social unit *A*, which has the highest coda repertoire similarity with its own social unit. (v) Conformism: calf *e* preferentially copies the coda types with higher frequencies of usage, here the three codas commonly performed by the adults. (vi) Symbolic marking: calves *f* and *g* were born in different social units, which have a specific subset of codas (‘symbol') that all members always perform to mark the identity of the unit (the sequences of red codas). Both calves copy codas from other adults, but also deliberately copy their units' ‘symbols'. (**b**) Oblique social learning (iii) and the additional effects (iv–iv) occurred at the three social levels. (vii) Social unit: calf agents copy only from agents of their own social unit. (viii) Predefined clans: simulation started with predefined clan labels and calves copy from any agent inside of its predefined clan. (ix) Population: calves copy from any agent in the population. In all scenarios, calves had a low individual learning probability (replacing 1 random coda type by a random frequency) per year.

**Figure 3:**
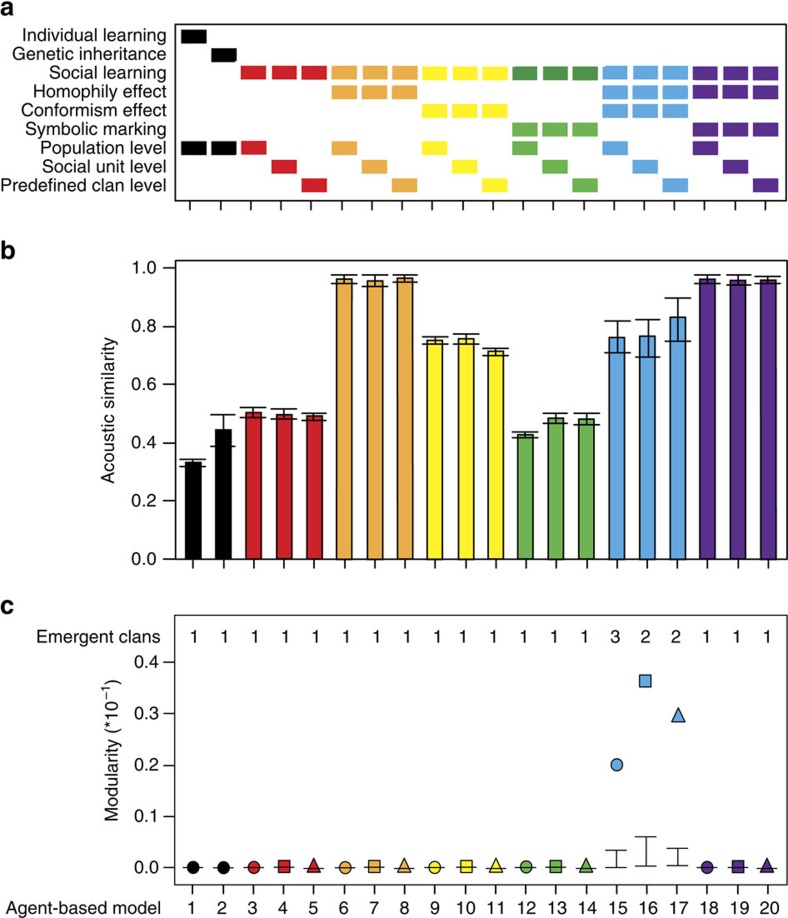
Coda repertoire similarities and clan partitioning across simulated scenarios. (**a**) Agent-based models (ABMs) differed in how a coda was transmitted (individual learning, genetic inheritance and social learning), if there was any transmission biases (homophily, conformism and symbolic marking), and the social level at which the transmission operated (population, social units and predefined clans). Columns represent ABMs and filled cells represent the presence of the model features (transmission mechanisms, biases and social levels) indicated in the rows. Colour code denotes similar transmission mechanisms operating at different social levels. (**b**) Average coda repertoire similarity of all emergent social units. Whiskers represent s.d. (**c**) Modularity (*Q*-values) of the resultant acoustic networks from each ABM. Significantly high modularity values (*P*<0.001) fall outside of the 95% confidence intervals (whiskers) generated by a theoretical model (1,000 replicates) and indicate the emergence of vocal clans, that is, modules of highly connected social units due to high coda repertoire similarity in the acoustic network. Number of emergent vocal clans is listed on the top of the plot; symbol shapes denote the social level where the transmission mechanisms operated (circle: population; square: social unit; and triangle: predefined clans).

With agent-based models (ABMs) empirically parameterized (Fig. 2; Supplementary Methods), we simulated 20 scenarios (Fig. 3a) to test which transmission processes of coda types between individuals—individual learning, genetic inheritance, pure and biased oblique social learning—could split individuals into sympatric clans with similar acoustic behaviour, as observed empirically (Fig. 1). We focused on two high-level attributes of the simulated data: similarity of coda repertoires among social units, and the emergence of modules of social units with distinct repertoires.

The similarity of coda repertoires of social units varied considerably across the 20 simulated scenarios (Fig. 3b), which differed in how and at which social level the codas were transmitted (Fig. 3a). Scenarios with individual learning (ABM 1), genetic inheritance (ABM 2) and pure oblique social learning (ABM 3) yielded the lowest acoustic similarities. Neither vertical transmission (when agents received the same codas from their mothers, mimicking genetic transmission and/or mother-to-offspring learning) nor pure social learning of codas (when agents copied coda types from each other) was sufficient for coda repertoires to diverge between social units. Instead, differences among individuals propagated over time and did not significantly deviate from the structure seen with the null ABM with just individual learning of codas (when coda types and frequencies of use were randomly assigned to agents; ABM 1).

However, the coda repertoires of social units tended to become more similar and less variable when social learning was biased via two distinct effects: homophily (agents preferentially copying adults whose social units have similar repertoires to their own; ABMs 6–8) or conformism (agents preferentially copying the most common coda types; ABMs 9–11). By introducing symbolic marking (when agents of different social units were assigned to specific subsets of codas; ABMs 12–14), simulations had more distinct starting points, which were maintained over time reducing similarity among social units' repertoires. While the combination of symbolic marking with homophily pushed all agents to a homogeneous repertoire (ABMs 18–20), the combined effect of homophily and conformism led to an overall high similarity, but with more variability among repertoires than other scenarios (ABMs 15–17; Fig. 3b).

**Figure 4:**
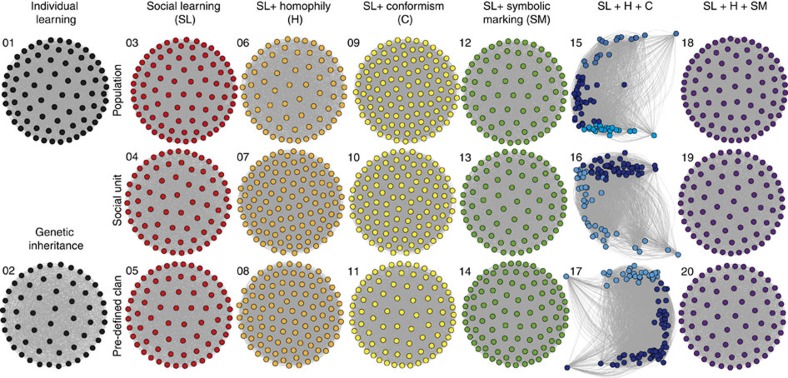
Acoustic networks simulated by the 20 agent-based models (ABMs). Nodes representing social units are connected by links representing coda repertoire similarity. Colour code indicates similar transmission mechanisms operating at different social levels across the ABMs, which differed in the coda transmission process (individual learning, genetic inheritance, social learning), in the presence, type and combination of transmission biases (columns: homophily, conformism, symbolic marking), and in the social level at which the transmission operated (rows: population, social units, and predefined geographically-segregated clans). ABMs 1 and 2 represent the null agent-based models with no social learning, but individual learning and genetic inheritance of codas, respectively. From ABM 3 to 20, all models contain social learning of codas, with and without biases as indicated. Distinct clans (modules in blue shades) emerged only when codas were transmitted by social learning (SL) biased by conformism (C) and homophily (H) operating in tandem at the population (ABM 15), social unit (ABM 16) and pre-defined clan levels (ABM 17). In all remaining scenarios, the acoustic network resembled the null models (ABMs 1–2), with no distinct clans.

Sperm whale clans exhibit distinct coda repertoires in the wild. This pattern was evident in only three simulated acoustic networks, all of which included biased learning (Fig. 3c). Modules of highly connected social units representing clans emerged only when codas were socially learned with homophily and conformism in tandem, regardless of the social level in which they operated (ABMs 15–17; Fig. 4). In these three scenarios, the measure of clan partitioning (modularity) was significantly higher than the theoretical expectation and four orders of magnitude higher than the remaining scenarios (Fig. 3c; Supplementary Table 1). The more complex scenarios—with combinations of social learning, symbolic marking and homophily even starting with predefined geographically segregated clans (ABMs 18–20)—performed similarly to the simpler scenarios (ABMs 3–11) with almost zeroed modularity values. In consequence, the topology of the acoustic networks produced by these remaining scenarios was almost completely connected, with no emergent clans (Figs 3c and 4), resembling the networks produced by the null models with no social learning (ABMs 1–2). While clans did not emerge in the complex scenarios due to the convergence of the repertoires into a single and homogeneous one, in the simpler scenarios clans did not emerge due to the overall low similarity among social unit repertoires.

### Robustness of the emergence of clans

Our findings emphasize the importance of transmission mechanisms, particularly biased social learning, on similarity and divergence of acoustic behaviour of sperm whales, showing that behavioural learning can create social hierarchies in sympatry. We accounted for the effect of movements of individuals—while respecting the structure of the sperm whale societies with nearly-permanent social units—in coda transmission by replicating the transmission mechanisms in the three social levels relevant for the sperm whales (social units, predefined geographically segregated clans and population). Similarity and modularity patterns were consistent across the different social levels where the transmission mechanisms operated (Fig. 3b,c) indicating that different degrees of population mixing had negligible effects on the emergence of clans.

Furthermore, clan emergence was robust regardless of initial conditions and across varying parameterizations (Supplementary Methods). A sensitivity analysis yielded similar outputs (Supplementary Fig. 1): only the very same three scenarios with biased social learning of coda types (ABMs 15–17) yielded significant modules in the acoustic networks, regardless of model parameterization. Therefore, the proportion and dispersal of potential tutors (adult agents) and learners (calf agents) and copying errors or innovations as source of cultural traits (coda types) are shown to have a minor effect on the diversity of cultural traits (coda types) and thus the emergence distinct cultural clans of whales (Supplementary Note 1). Moreover, the metric of clan emergence was robust to variation in the sampling of acoustic similarity among social units (Supplementary Methods). For all of the 20 ABMs, modularity was high, stable and consistent across the range of possible weights for a link (coda repertoire similarity) between nodes (social units) in the simulated networks (Supplementary Fig. 2; Supplementary Note 2).

## Discussion

Our empirical findings and simulations combined reveal how social levels of sperm whales are nested and point to cultural transmission as the most likely mechanism giving rise to the upper social level of their multilevel society. Whale clans are based on learning of communication signals, and biased learning may be necessary to generate sympatric culturally driven social tiers. By modelling the processes that give rise to the complex and highly structured social system of sperm whales, we show that key processes attributed to human culture may not only be present in non-human societies, but also likely created the social structure we observe in sperm whales. While ecological, cognitive, and time constraints and benefits^11^,^12^,^13^ may delineate the lower sperm whale social level (social units) as in other multilevel societies^7^, we suggest that the process that has produced the higher level (clans) is information flow.

In small multilevel human societies, the flow of information, materials, energy, genes and/or culture among individuals plays an important role in regulating the quantity and quality of social relationships^5^,^46^. Would the flow principles of a two-dimensional terrestrial world create similar additional selective forces for sociality in the three-dimensional marine environment^7^? While predation and resource availability are thought to be basal for sociality in both environments, marine resources generally tend to be more dispersed and less predictable in space and time^47^,^48^, making it logistically impossible for marine mammals to defend or transfer resources among individuals (though they are sometimes shared). We hypothesized that, in this situation, information is the primary resource that can be stored and transferred among selected companions; the way such information flows can subsequently shape the structure of their society^16^. Take for example the knowledge of how to manipulate a certain food resource: it can certainly flow within a population through social learning among individuals, but sometimes not among all of them, which demarcates subsets of the society with distinct behavioural repertoires^28^,^49^. In addition to foraging and movement behaviours^50^, sperm whales, and other cetaceans, can learn acoustic communication signals from each other^41^. In the aquatic environment, where sounds spread particularly well, acoustic communication—the likely function of codas^51^—may help maintain group cohesion, reinforce bonds, aid negotiations and collective decision-making^52^,^53^. Since vocal learning in cetaceans is an output of complex social behaviour and may assist the maintenance of multiple social relationships^41^, we asked if it could trigger the formation of the vocal clans.

This question is logistically unable to be answered using experimental manipulations or observational studies (which would not provide a mechanism), making an agent-based simulation an ideal approach. Our simulations suggest that clans are unlikely products of stochastic processes, such as genetic or cultural drift. The simulated acoustic behavioural segregation seems not to be a collection of individual innovations diverging over time, or an artefact of genetic transmission. Whereas vertical, mother–offspring, social learning can establish and maintain behavioural traits in some cetacean populations (for example, tool use and foraging tactics in bottlenose dolphins)^49^,^54^, our models suggest that oblique social learning is necessary to promote clan-like vocal repertoires among sperm whales.

Still, social learning alone was not enough to segregate social units into clans, and our vocal clan recipe needed additional ingredients. Social learning is susceptible to biases^22^, which affect flow of information and potentially the emergence of cultural patterns. The theoretical expectation is that unbiased transmission can only lead to marked cultural differences among allopatric, strictly isolated groups of individuals^55^. Yet, how are different coda repertoires maintained in the same Pacific waters? Our results suggest that the answer may be in biased transmission, which can maintain similarities within, and disparities between, sympatric cultural groups through time^36^,^55^. By means of feedback between homophily and social influence^17^,^18^, individuals who behave similarly preferentially associate and learn from one another, increasing their behavioural similarity. This process breeds relationships among like-minded individuals, and simultaneously tends to dissolve relationships between individuals with distinct behaviour. As seen in our simulations, this effect is leveraged when individuals are conformists and disproportionately learn the most common behavioural traits^19^,^56^. Combined, behavioural matching and majority-biased transmission can promote a segregation of individuals into behaviourally distinct groups^16^,^26^.

This is the case in our simulations: when whales were more prone to learn the most common coda types from those who already had similar coda repertoires, clans of social units with distinct acoustic repertoires emerged and matched the pattern observed empirically. Increasing behavioural homogeneity with some peers, and so reinforcing social differentiation and cultural boundaries between subsets of the population, can be particularly striking in sympatric large toothed whale societies. As with Pacific sperm whales, killer whales (*Orcinus orca*) can display remarkable intrapopulation behavioural segregation, seemingly marked by an intricate system of pulsed calls for communication^9^,^57^. These calls can change over space and time, but tend to do so in a way that preserves the differences between pods^58^, suggesting that acoustic signals can allow pods to distinguish themselves.

Our findings highlight the contribution of behavioural transmission mechanisms, as opposed to purely demographic or spatial factors, in the emergence of the sympatric cultural groups. Other factors, such as population size and proportion of tutors as source of cultural traits, age distributions, dispersal rates or copying errors/innovations^34^,^35^,^59^,^60^ played a minor role and had no substantial impact on the emergence of clans of sperm whales. In addition, the complete spatial isolation into predefined geographically segregated clans^44^, with members learning only within their clans, did not produce the acoustic divergence required to split the population into distinct dialects. This shows that multilevel social structures can arise even in the absence of spatial and temporal heterogeneity, implying that learning mechanisms may have more influence in driving social structure than previously thought.

In conclusion, sperm whales are distinctive among multilevel animal societies with the higher social level produced by biased cultural transmission. By modelling the evolution of the repertoire of acoustic communication signals, we were able to overcome the logistical impossibilities of field experiments in the vast spatiotemporal scales that are relevant for sperm whales, and show that the sympatric behavioural segregation that delineates the sperm whale clans is not controlled by genes, nor genetic or cultural drift. Our empirically-founded models incorporating population dynamics and multiple transmission mechanisms indicate that learning coda types plays a crucial role in promoting similarity in acoustic behaviour, and, more strikingly, that biases in social learning are required to split the sperm whales into sympatric clans with distinct dialects observed in the wild. We suggest therefore that empirical clans have emerged like the simulated ones: as a cultural segregation. While transmission biases drive culture and social structure in humans^36^, there is much debate about whether or not they are exclusive features of human culture^61^. Providing evidence that the processes generating the complex and diverse cultures in human populations could also be at play in non-human societies is a crucial step towards evaluating the contrasts and convergences between human and non-human cultures.

## Methods

### Empirical data and social levels definition

Sperm whales groups were tracked visually and acoustically, day and night, during 2 to 4–week research trips between 1985 and 2003 in the Eastern Pacific Ocean, mainly off the Galápagos Islands (summarized in ref. 39). Three nested social levels were evident within the Pacific sperm whale society: individuals, social units and vocal clans. Individuals were identified by photographic records, comparing the natural markings on the trailing edge of tail flukes^62^. Social units were sets of about 12 individuals^39^ that live and move together for years, delineated using association indices on long-term photo-identification data^45^. Clans were sets of social units with high similarity in their coda repertoires, the stereotyped patterns of clicks used for communication^40^. Codas were recorded with hydrophones and repertoires were assigned to the social units whose members were photo-identified within 2 h of the recording and had at least 25 codas recorded^30^. The social units' repertoires were compared to define the best partition of social units with distinct repertoires into clans^30^. Clans are estimated to contain many social units and several thousands of members, based on the estimated abundance of sperm whales in the Pacific and number of clans^30^. Social units are typically found in behaviourally coherent groups with other units from their own clan, but never with units from different clans even though sperm whales from several clans may use the same waters^30^.

### Agent-based modelling

We simulated the interactions of multiple individual whales using an agent-based modelling framework (ABM) to test whether the clan structure observed in the sperm whale society could arise from evolving vocal behaviour. The ABMs were built in R^63^ based on empirical parameters (Supplementary Methods), and are described according to the Overview, Design concepts and Details protocol^64^ as follows:

- *Purpose*. The models test which transmission mechanisms for acoustic behaviour, if any, can give rise to clans of social units of sperm whales with distinct acoustic repertoires, and explain the multilevel social structure observed empirically.
- *Entities, state variables and scales*. The models have one kind of agent that behaves under realistic life-history parameters (empirical support for model parameters available in the Supplementary Methods): female whales that learn coda types at ages of 0 to 2 year old. Because male sperm whales lead quasi-solitary lives and rarely produce codas^62^,^65^ they were not represented in the models. The agents are characterized by their age (years), their coda repertoire (a vector of frequencies of use of different coda types), and which social unit and vocal clan they belong to (nested categorical variables). The models were explicitly temporally-structured and implicitly spatially-structured. However, we accounted for different levels of population mixing (and so implicitly for individual movements), with coda transmission operating among individuals of three different social levels (see ‘d'). Simulations lasted for 700 time steps (years).
- *Process overview and scheduling*: During each time step, biological processes occurred in the following order: birth, coda repertoire composition and changes (at ages up to 3-year old), social unit membership change (or not) and death. Calves have a high probability of staying with their natal group, and migration of individuals among social units is rare^45^; thus nearly-permanent and nearly-matrilineal social units are an emergent property. In these respects, the models mimic several transmission processes characteristic of some socially complex species. We started with two null agent-based models without social learning: in one the agents only learn their codas individually; in the other they receive their mothers' coda repertoire, representative of genetic inheritance (as well as stable vertical–cultural transmission). We then simulated a total of 20 complementary scenarios with combinations of oblique social learning of coda types and transmission biases operating at the three different social levels (see ‘g').
- *Design concepts*. There are two emergent properties of the interactions among agents: social units (sets of females and their offspring who stay together during the simulated time) and vocal clans (sets of social units with highly similar vocal repertoires). Social units emerge in all models and vocal clans can be predefined (see ‘g'), or may emerge. All demographic processes were modelled with demographic stochasticity and parameterized from empirical studies (see Supplementary Methods). Birth rates were age-specific, and mortality rates were density- and age-dependent (calf agents had higher probability of dying than adult agents^66^), and migration rates of individuals between units were low and decreased with age. The main process of interest modelled is changes in individual coda repertoires, that is, in frequencies of use of coda types. Each agent has a repertoire represented by a vector with 62 elements denoting continuous absolute frequencies of different coda types from 0 (absent) to 100 (always performed coda type) (Fig. 2a) (details in Supplementary Methods). Calves compose repertoires at early ages (although precise age is inherently difficult to estimate empirically; Supplementary Methods). Repertoire composition was represented by calf agents replacing some coda types and frequencies once a year, while at ages of 0, 1 and 2. At age 3, all agents' repertoires were fixed. Depending on the sub model (see ‘g'), the repertoire change occurred according to one of the three main transmission processes (see Fig. 2a): (i) individual learning—calf agents compose their own coda repertoires, that is, are assigned to random coda types and frequencies drawn from uniform distribution ∈[0,100]; (ii) genetic inheritance (which also represents vertical social learning)—calf agents receive their mothers' coda repertoires; and (iii) oblique social learning—calf agents copy coda types and frequencies from adult agents, of different generations, kin-related or not. For the models with oblique social learning, the three following effects were included: (iv) homophily—calf agents preferentially copy codas from adult agents of social units with the highest repertoire similarity with the calf's social unit's repertoire. (The homophily effect posits that behaviourally similar individuals tends to interact more often^17^; since social learning occurs during social interaction, the homophily effect on learning can be represented as individuals with similar behaviour learning preferentially from each other.); (v) conformism—calf agents disproportionately copy the most common coda types; and (vi) symbolic marking—all agents of a given social unit are assigned to a random sequence of six coda types with frequency of usage 100 (a ‘symbol') at time *t*=1, to mark the identity of their units; all calf agents from *t*=1 deliberately copy the ‘symbol' of the unit to which they belong. To account for different degrees of population mixing, we replicated the models with oblique social learning and additional effects (iii–iv) across the three levels of the sperm whale society: social unit, predefined clans and population (Fig. 2b): (vii) social units—calf agents randomly copy codas from agents of their own social unit; (viii) predefined clan—agents were arbitrarily assigned to three clans and calf agents learned only from adult agents of their own clan. (Since clan partition could be driven by non-learning mechanisms, we simulated the pre-existence of clans representing geographically-segregated clans such as those that seem to occur in the Atlantic where acoustic variation is driven by spatial isolation^44^. We refer to these as ‘predefined clans' as opposed to the ‘emergent clans' that may arise in the simulations due to acoustic similarity.); (ix) population—calf agents learn from any agent in the population. We combined transmission mechanisms, effects and social levels in a total set of 20 ABMs (see ‘g', Fig. 3a). At the end of each simulation, we observed the number and size of social units and vocal clans and how similar their coda repertoires were (methods below).
- *Initialization*. Simulations were initialized with the following parameters based on empirical data (details, justification and references in Supplementary Methods). At the first time step, year *t*=1, all simulations started with a population of *N*_0_=1,000 agents, to which ages were randomly assigned from a negative exponential distribution (so the initial population was mostly young, with ages typically varying from 0- to about 70-year old), and social unit membership labels were assigned with equal probabilities. Each agent received an empty vector of 62 elements (that is, coda types) representing their coda repertoire. For each agent, half of the elements in its coda repertoire vector were randomly selected to receive an absolute frequency of usage from a uniform distribution ∈[0,100] (absent coda type=0; always performed=100). Agents are considered calves when they are 0-, 1- and 2-year old, during which changes in the coda repertoire occurred. Adult female agents became sexually mature after 9-year old, stopped reproducing after 41-year old, and lived 70 years on average. Population was modelled density dependent, with age-dependent reproduction, mortality and migration rates, such that the population fluctuated around the carrying capacity (*N*_*0*_) over time. The initial number of social units was based on the initial population size (*N*_*0*_) and empirical average unit size in the Pacific (about 12 members). Social units split in half when double the maximum initial unit size. Calf agents remained in the mother's social unit since they highly depend on their mothers, and adult agents had low probability of randomly migrating to other social units during their lives (*c=*0.05). Repertoire changes were represented by replacement of frequencies of coda types and occurred three times for each agent (repertoires were fixed at the age of 3 years). Newborn agents started the simulation with empty coda repertoires; each simulated year, all calf agents changed their coda repertoires under one of the three main mechanisms—with additional effects or not—operating at one of three social levels (see ‘g', Fig. 2). In all models, calf agents also had low individual learning rate (*ilearn*=0.02), that is, each year replacing the frequency of one coda type (62 codas × 0.02≈1) chosen at random by a frequency drawn from a uniform distribution ∈[0,100], which accounted for random learning errors or deliberate innovations^60^. Supplementary Fig. 3 illustrates the population output measures of a typical simulation.
- *Input*. The models have no external input data, but initial parameters differed in sub models.
- *Sub models.* We created a total of 20 sub models (Fig. 3a), all of which have the same structure but differ in the way calves compose their coda repertoires (Fig. 2). In the first null agent-based model (ABM 1), calf agents learn their coda repertoire only through individual learning. In the second null agent-based model (ABM 2), calves receive the exact repertoire of their mothers, mimicking genetic or vertical–cultural transmission of coda repertoires. In all the following models (ABMs 3–20), calves change repertoires with oblique social learning, some with combinations of the three transmission biases: homophily (ABMs 6–8 and 15–10); conformism (ABMs 9–11 and 15–17); and symbolic marking (ABMs 12–14 and 18–20). Oblique social learning and its biases occurred within social units (ABMs 4, 7, 11, 13, 16 and 19), across social units of the same predefined clans (ABMs 5, 10, 14, 17 and 20) and in the entire population (ABMs 3, 6, 9, 12, 15 and 18).

### Coda repertoire similarity

The empirical repertoires of the social units were compared based on the inter-click intervals of each coda using an averaged multivariate similarity metric^30^. Because in the ABMs we simulated frequencies of usage of coda type—and not the inter-click intervals of each coda—we compared repertoires of each pair of simulated social unit with the weighted Bray–Curtis index between the average frequency of usage of codas of all agents of these units. We adjusted the index to represent similarity, which ranged from 0 (completely different) to 1 (exactly the same repertoire). We detail the differences between empirical and simulated codas and repertoire comparison in the Supplementary Methods.

### Clan partition in empirical and simulated data

Clan partitioning in the simulated data was adapted from the original methods for vocal clan definition: the social units' coda repertoires were compared and the best partition into clans was based on the repertoire similarity^30^. While the original approach included hierarchical clustering, we used the network formalism to depict social units (nodes) connected by similarity of coda repertoires (links) and modularity to define the emergence of clans (see below). To allow for direct comparisons, we reanalysed the empirical social and acoustic data^30^,^45^ with the same network framework. First, we built a social network of photo-identified individuals (nodes) connected by the strength of social relationships (links), that is, the proportion of time individuals were seen together^45^ estimated by the half-weight association index. We then overlapped the empirical acoustic network, in which the social units (nodes) were connected by the similarity in their averaged coda repertoires (links).

For both empirical and simulated data, vocal clans were defined by modules in the acoustic networks, that is, subsets of nodes (social units) that are highly and strongly linked within each other (by acoustic similarity) and weakly linked with the rest of the network. We searched for the best module partition using the Walktrap algorithm^67^, which is based on the assumption that random walks in a network will tend to get ‘trapped' inside strongly connected modules. More specifically, this algorithm uses an agglomerative approach to form modules, using a distance metric based on the probability of a random walk from node *i* to node *j*. Hence, nodes belonging to a given module will share similar probabilities of going to nodes outside their module. To the resultant hierarchy of modules, the largest increase ratio of the total distance is used to infer the best partition into modules. Subsequently, we assigned a value to this partition using the weighted version of modularity metric *Q*^68^:


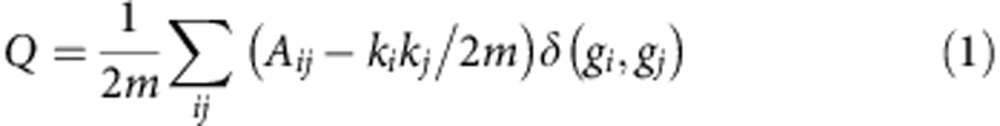


where *A* is a weighted adjacency matrix, with elements representing the acoustic similarity between social units, 
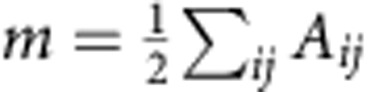
 is the weighted number of links, *k*_*i*_ is the weighted degree of node *i* and 
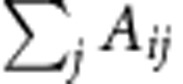
 and *g*_*i*_ gives the label of the module (herein clan) the node (herein social unit) *i* belongs to.

The significance of clan emergence, both in empirical and simulated data sets, was assessed comparing the modularity *Q*-values to a benchmark distribution generated from 1,000 theoretical networks. We created theoretical networks with the same size (number of nodes, that is, social units), same link weight distribution (that is, acoustic similarity) and connectance (proportion of realized links) using a model that randomizes the link weights among nodes^69^. Clan emergence was considered significant whenever the modularity *Q*-values of the observed acoustic networks were outside of the 95% confidence intervals of the benchmark distribution.

### Sensitivity analysis and robustness of clan emergence

The parameters and initial conditions of the ABMs were grounded on empirical evidence (Supplementary Methods) and fixed across scenarios to allow for directly comparison of learning strategies without any confounding influence of other changing parameter values. To evaluate whether the observed partition of social units into clans was robust to varying the initial conditions in the models, we performed a sensitivity analysis of the 6 initial demographic and 2 learning parameters that were common to all of the 20 ABMs (population size and carrying capacity, reproductive age, migration rate, mortality rates, age distribution, initial average social unit size, individual learning rate and coda repertoire size; full description in Supplementary Methods). We ran each ABM changing a single parameter value at a time to two extreme parameter estimates of a biologically meaningful range (Supplementary Table 2) and calculated modularity and 95% confidence intervals with the theoretical model described above. Specifically, we tested whether changing the ABMs initial setup would still yield emergence of clans in the scenarios with biased social learning (ABMs 15–17); and, conversely, whether clans would emerge in the rest of the scenarios in which they originally have not emerged (ABMs 1–14 and 18–20; see Figs 3c and 4).

In addition, we evaluated the robustness of the metric for clan partitioning (modularity) by bootstrapping the links of the 20 simulated acoustic networks (Supplementary Methods). The simulation of coda repertoires by the ABMs represented a complete sampling, in the sense that all codas of all agents of all social units were recorded and compared. This is clearly not the case for the empirical data, in which field logistics inherently yield incomplete sampling of the social units' coda repertoires. To make empirical and simulated data more comparable and assess whether the modularity patterns in the simulated data were consistent in subsets of the simulated data, we resampled the acoustic network weighted links (that is, coda repertoire similarity between social units) with replacement (bootstrap, 1,000 iterations) and calculated the weighted modularity with increasing sampling, from 5 to 100% with increment of 5% of the links at a time.

## Additional information

**Accession codes** Agent-based models and data are available in the R package *balabm* v.1.1 (Supplementary Methods).

**How to cite this article:** Cantor, M. *et al*. Multilevel animal societies can emerge from cultural transmission. *Nat. Commun.* 6:8091 doi: 10.1038/ncomms9091 (2015).

## Supplementary Material

Supplementary Information Supplementary Figure 1-3, Supplementary Tables 1-2, Supplementary Note 1-2, Supplementary Methods and Supplementary References.
