# Supplementary material for: Multilevel animal societies can emerge from cultural transmission
Source: Nat Commun. 2015 Sep 8;6:8091. doi: 10.1038/ncomms9091 (PMC4569709; doi:10.1038/ncomms9091)
Supplement: Supplementary Information — Supplementary Figure 1-3, Supplementary Tables 1-2, Supplementary Note 1-2, Supplementary Methods and Supplementary References. [file ncomms9091-s1.pdf]

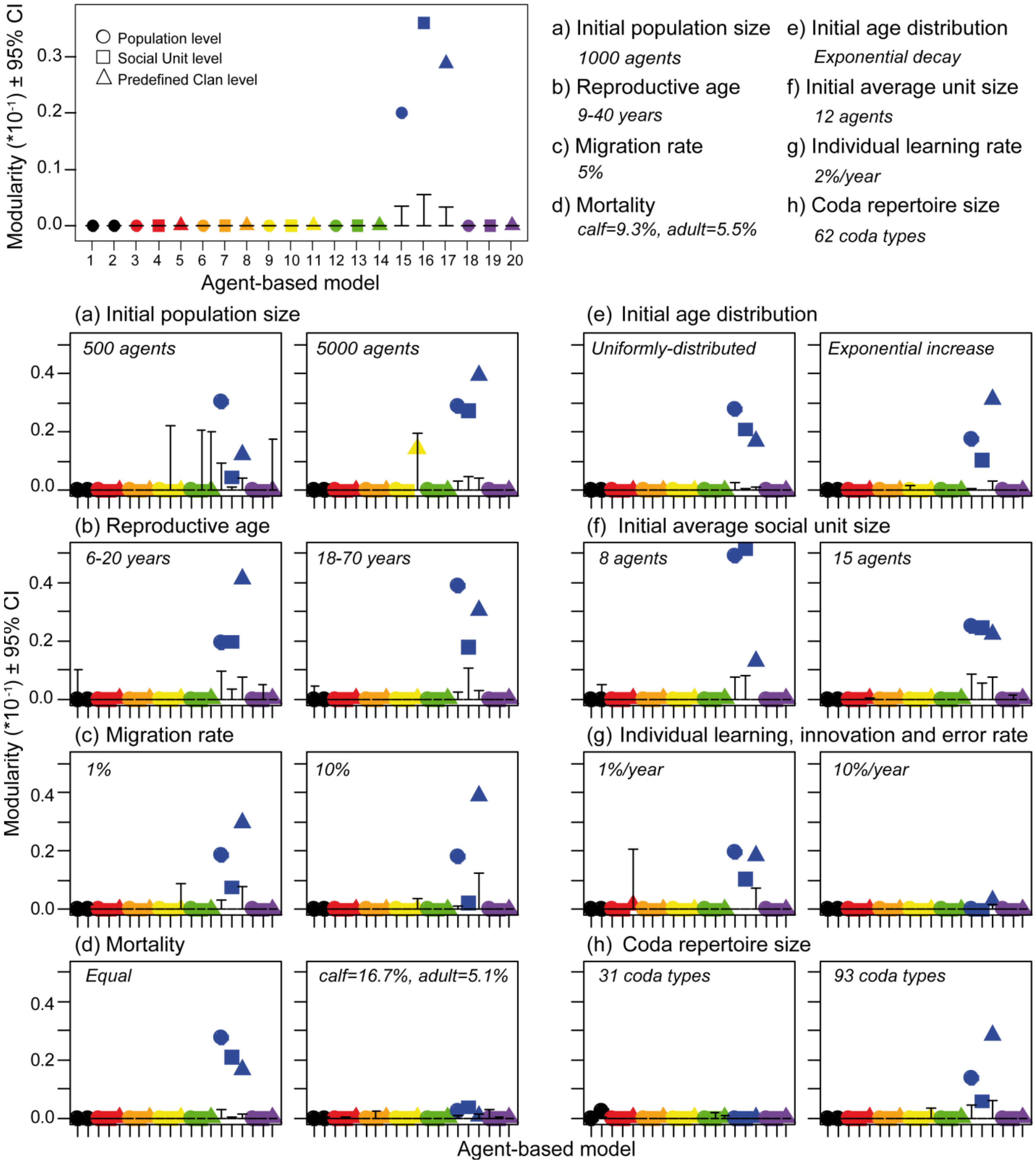

Supplementary Figure 1. Sensitivity analysis of parameterization and initial conditions in the emergence of clans in the 20 simulated scenarios. Clan emergence is evaluated by the weighted modularity *Q*-value (y-axis) in each of the acoustic networks simulated by the 20 agent-based models (x-axis). Color code denotes similar transmission mechanisms of coda types among agents operating at

different social levels (see Fig. 3A, main text); shapes represent the social level at which the transmission operated (circle: population, square: social unit, circle: predefined geographical clan); whiskers represent the 95% confidence intervals (CI) estimated by an appropriate theoretical model, based on 1000 iterations and 10 replicates for each model. Significant *Q*-values, those falling outside of the 95% CI of the benchmark distribution ($P < 0.001$), indicate a reliable partition in the acoustic networks, i.e. a modular topology with subsets of social units (nodes) more strongly connected with each other than with the rest of the network (see Fig. 4, main text). The top plot illustrates our main results (Fig. 3C, main text), followed by their initial parameters values. In each of the 16 new scenarios below (a-h), a single parameter was changed at a time for all 20 models with values representing the extremes of a biologically meaningful range (the parameter value is indicated in the top left of the plot). Note that each small plot resembles very much the main pattern shown in the top plot, indicating that the clan emergence presented in the main text is robust to variation in the parameters and initial conditions.

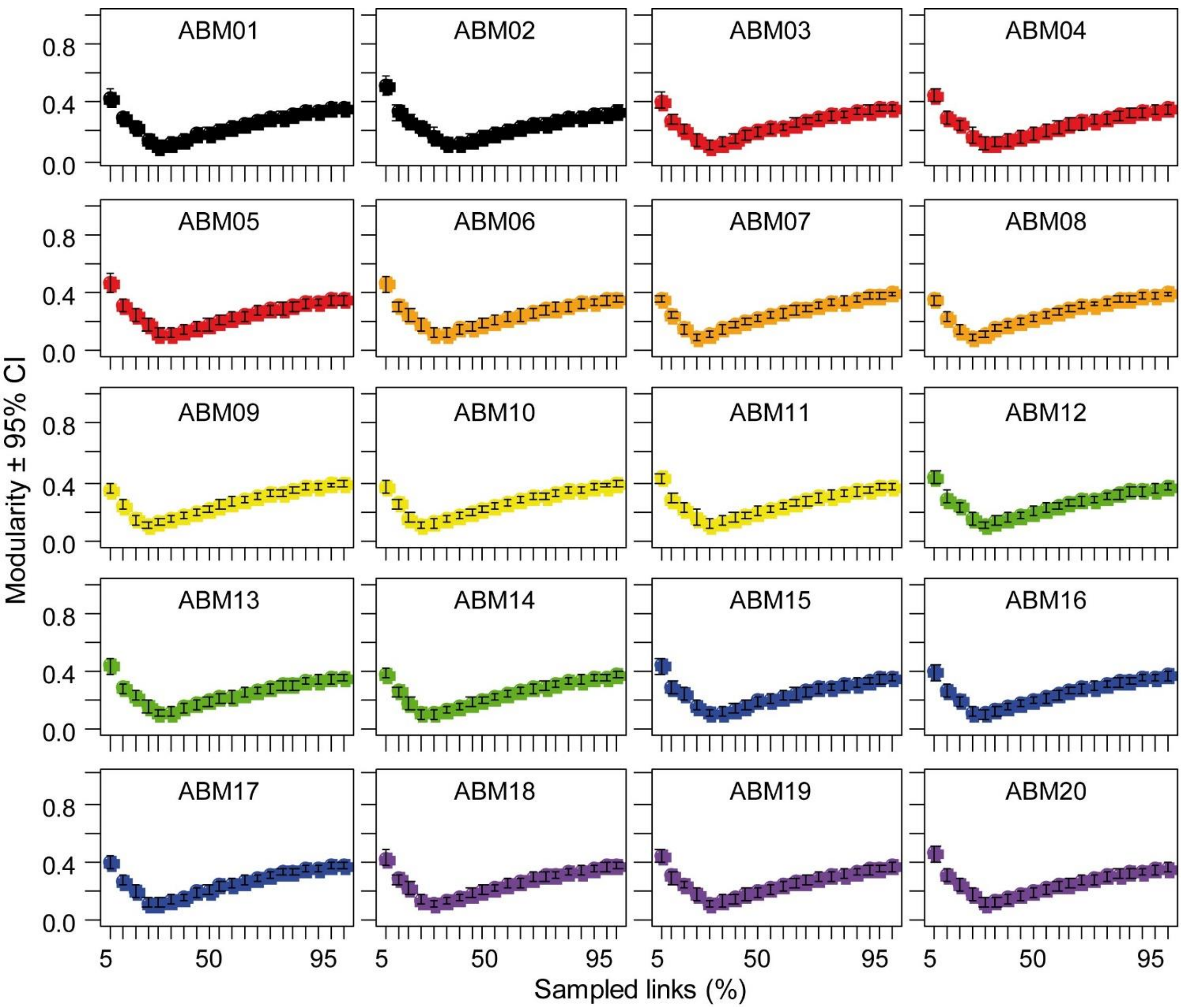

Supplementary Figure 2. Sensitivity analysis of the weighted modularity metric along a gradient of increasing sampling of the link weights in the acoustic networks. The modularity metric defines a reliable partition in the acoustic networks (i.e. emergent clans) simulated by each of the 20 agent-based models (ABMs). Whiskers represent the 95% confidence intervals (CI) given by 1000 replicates in the bootstrap procedure (link weight resampling with replacement). Color code denotes similar transmission mechanisms of coda types among agents operating at different social levels (following Fig. 3A, main text). Note the strikingly similar pattern in all scenarios, conferring robustness to our chosen metric for clan partition across the range of possible weights for a link (coda repertoire similarity) between nodes (social units) in the simulated networks.

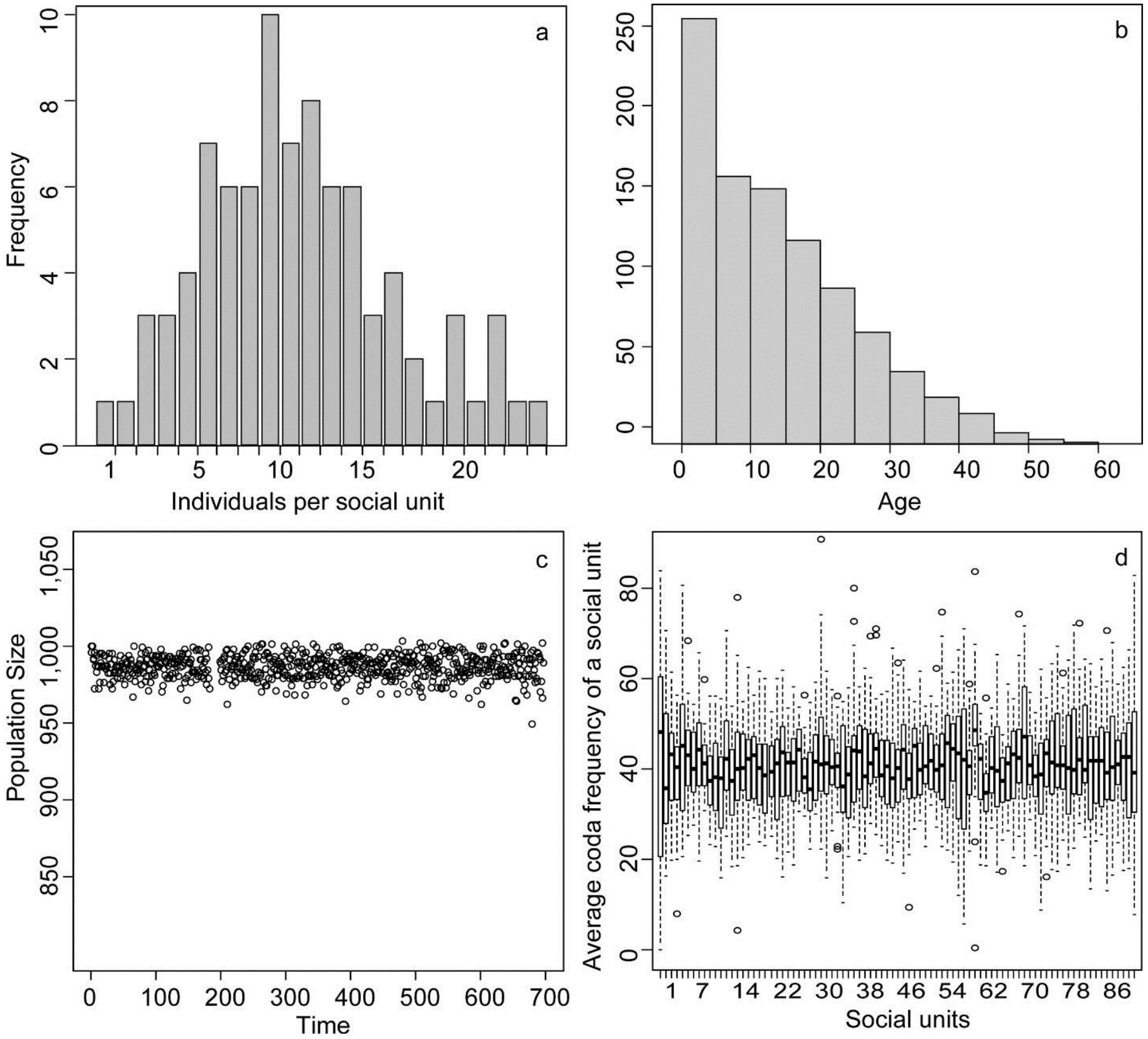

Supplementary Figure 3. Typical simulated dataset. Output of a representative simulated dataset, with social learning of coda types within social units (ABM04), after 700 simulated years: (A) Social unit size distribution (number of agents); (B) Age distribution (in simulated years, i.e. time steps in the simulation); (C) Population size over time (number of agents); (D) Coda frequency of all repertoires of all agents within each social unit. Each boxplot represents the frequency distribution of a social unit; boxes represent 1st and 3rd quantiles; black horizontal bar represent median; dashed whiskers represent minimum and maximum frequencies; circles indicate outliers.

Supplementary Table 1. Modularity results and averaged acoustic similarity in all 20 agent-based models.

ABM	Transmission process and level of operation	Modularity		Average acoustic similarity ± SD				
				Emergent social units		Emergent clans		
		Q	95% CI	within	between	#	within	between
01	Individual learning (IL)	0	0 – 0.6*10^{-8}	0.3301±0.0122	0.3297±0.0079	1	0.3301	-
02	Genetic inheritance (GI)	0.1*10^{-8}	0 – 0.8*10^{-8}	0.4405±0.0533	0.3902±0.0312	1	0.3913	-
03	Social learning (SL) at population level	0	0 – 1.7*10^{-8}	0.5026±0.0196	0.5017±0.0127	1	0.5026	-
04	Social learning (SL) at unit level	0	0 – 0.2*10^{-8}	0.4958±0.0170	0.4956±0.0120	1	0.4958	-
05	Social learning (SL) at clan level	0	0 – 0.4*10^{-8}	0.4874±0.0138	0.4875±0.0106	1	0.4872	-
06	SL+Homophily (H) at population level	0	0 – 0.9*10^{-8}	0.9592±0.0139	0.9545±0.0106	1	0.9592	-
07	SL+Homophily (H) at unit level	0	0 – 0.2*10^{-8}	0.9546±0.0196	0.9494±0.0142	1	0.9546	-
08	SL+Homophily (H) at clan level	0	0 – 0.5*10^{-8}	0.9613±0.0103	0.9570±0.0077	1	0.9575	-
09	SL+Conformism (C) at population level	0.2*10^{-8}	0 – 0.9*10^{-8}	0.7477±0.0122	0.7470±0.0101	1	0.7495	-
10	SL+Conformism (C) at unit level	0	0 – 0.9*10^{-8}	0.7538±0.0171	0.7536±0.0123	1	0.7538	-
11	SL+Conformism (C) at clan level	0.6*10^{-8}	0 – 0.7*10^{-8}	0.7094±0.0119	0.7086±0.0082	1	0.7086	-
12	SL+Symbolic Marking (SM) at population	0	0 – 0.3*10^{-8}	0.4246±0.0100	0.4253±0.0068	1	0.4246	-
13	SL+Symbolic Marking (SM) at unit level	0	0 – 1.0*10^{-8}	0.4821±0.0171	0.4813±0.0103	1	0.4821	-
14	SL+Symbolic Marking (SM) at clan level	1.0*10^{-8}	0 – 0.5*10^{-8}	0.4794±0.0199	0.4801±0.0127	1	0.4808	-
15	SL+H+C at population level	0.020	0 – 0.003	0.7625±0.0540	0.7494±0.0491	3	0.7343±0.0684	0.7302±0.0054
16	SL+H+C at unit level	0.036	0 – 0.006	0.7582±0.0639	0.7437±0.0498	2	0.7183±0.0852	0.7115±0.0046
17	SL+H+C at clan level	0.029	0 – 0.003	0.8210±0.0730	0.8089±0.0581	2	0.8191±0.0112	0.8189±0.0056
18	SL+H+SM at population level	0.9*10^{-8}	0 – 1.7*10^{-8}	0.9581±0.0152	0.9531±0.0110	1	0.9538	-
19	SL+H+SM at unit level	0	0 – 0.7*10^{-8}	0.9546±0.0179	0.9479±0.0148	1	0.9546	-
20	SL+H+SM at clan level	0.5*10^{-8}	0 – 1.5*10^{-8}	0.9567±0.0142	0.9509±0.0103	1	0.9524	-

For each agent-based models (ABM01-20), modularity Q ± 95% confidence intervals (CI) calculated with theoretical models, and averaged coda repertoire similarity ± standard deviation (s.d.) within and between social units and emergent clans are presented. ABMs are composed by a transmission process (individual learning, genetic inheritance, social learning (SL)), with/without a transmission bias (homophily (H), conformism (C), symbolic marking (SM)), occurring at three different social levels (population, social unit, predefined geographically-segregated clan).

Supplementary Table 2. Parameters tested in sensitivity analysis of the agent-based model parameterization and initial conditions.

Parameter or starting condition	Lower estimate	Chosen condition	Upper estimate
a) Starting population size and carrying capacity (agents)	N_0 = 500	N_0 = 1000	N_0 = 5000
b) Reproductive age (years)	6 to 20	9 to 40	18 to 70
c) Migration rate among social units (agents year^{-1})	c = 0.01	c = 0.05	c = 0.10
d) Mortality probability (year^{-1})	Uniform (equal to all agents)	Age-dependent[1] (M_{calves} = 0.093, M_{adults} = 0.055)	Age-dependent[2] (M_{calves} = 0.1679, M_{adults} = 0.0505)
e) Initial age distribution (years)	Normal distribution (all ages are equally probable)	Exponential decay (more younger, less older agents)	Exponential increase (more older, less younger agents)
f) Initial average social unit size (agents)	*tvu* = 8	*tvu* = 12	*tvu* = 15
h) Individual learning rate (year^{-1})	*ilearn* = 0	*ilearn* = 0.02	*ilearn* = 0.10
g) Repertoire length (coda types)	*nCodas* = 31	*nCodas* = 62	*nCodas* = 93

Tested parameters were common to all of the 20 agent-based models. Three conditions for each parameter were tested: a lower extreme estimate, the chosen condition, and an upper extreme estimate.

Supplementary Notes

Supplementary Note 1. Collectively, the sensitivity analysis evaluated the robustness of the clan partition across various demographic, learning, and coda-specific parameterizations (see Sensitivity analysis of parameterization and initial conditions in the agent-based models, Supplementary Methods). The different effects considered were the proportion of potential tutors (adult agents) as source of cultural traits (coda types)[3], their dispersal rates[4,5] as seeding of copying errors and innovations[6,7] between social units on the transmission of coda types among learners (calves), the diversity of cultural traits in the population and hence the emergence distinct cultural groups[8], here the clans of sperm whales. The results were very robust to changes in the initial conditions and parameterization (Supplementary Fig. 1). This is clear in two key ways. First, in all of the 16 cases with different initial conditions, there was no reliable clan partition in the models in which they originally have not emerged (ABMs 1-11, 13-14, 18-20). In these cases, modularity were zero or non-significant and 2 to 3 orders of magnitude smaller than in the cases where clans emerged (Supplementary Fig. 1). Second, clans emerged only in the very same three models with biased social learning of coda types (ABMs 15-17) in which we originally observed partition of the acoustic networks into modules (Fig. 4, main text).

Although the overall pattern is robust to the initial conditions, there were a few deviant situations (10%: 5 situations/(3 models*16 cases)) in which these three models that originally yielded clans did not produce them when the parameters were set to extreme estimates (Supplementary Fig. 1). These were when: a) individual learning rate was excessively high in ABMs 15 and 16; and c) coda repertoire length was excessively small in ABMs 15, 16 and 17. We interpret these findings as follow. First, with very high rate of individual learning, the social learning efficiency decreases. Such rates (10% per year) would represent very low fidelity in copying coda types and/or very high rate of innovation by calves. Either way, the overall diversity increases, dismantling possible homogeneity in coda repertoires within potential clans. Second, with very small coda repertoires (c), there are few coda types and consequently little room

for variation. When averaging the repertoires of all individuals of social units, the overall diversity decreases, leading to the convergence of all units' repertoires and consequently to no partition into clans.

Supplementary Note 2. Our sensitivity analysis of our metric for the clan partition (see Sensitivity analysis of clan partition, Supplementary Methods) suggests that the clan emergence is robust to the variation in the averaged acoustic similarity among social units (acoustic network links) (Supplementary Fig. 2). In all the 20 agent-based models, the pattern was strikingly similar: modularity values were high, stable (with small 95% confidence intervals) and consistent from 5 to 100% of the sampled links (despite an overall decrease around 30%). This confers robustness to our chosen metric for clan partition across the range of possible weights for a link (coda repertoire similarity) between nodes (social units) in the simulated networks.

Supplementary Methods

The *balabm* R package. Agent-based models are available in the R package *balabm v.1.1*, which is deposited open-sourced at the Bitbucket repository https://bitbucket.org/maucantor/balabm. The package also makes available the empirical data used in this work.

1. Installation: The package can be installed from the repository:

   a. Launch *R*. Install and/or load the package '*devtools*' with the command:

   if(!require(devtools)){install.packages('devtools'); library(devtools)}

   b. Install and load '*balabm*' with the command:

   install_bitbucket("maucantor/balabm"); library(balabm)

2. Running the models.

   a. Browse your local machine for the folder where *R* was installed and find the folder */library/balabm.*

   b. The folder */abm/* contains the scripts for each of the ABM (Check README.txt). Copy the content of this folder to your working directory.

   c. Open the *R* scripts for the agent-based models in *R* (or *RStudio*) to run the models.

When using these models or data, please cite the R package along with the original paper:

citation("balabm")

Empirical support for the agent-based models. We built agent-based models based on the following empirical socioecological findings:

a. Social structure parameters

i. Female and immature sperm whales are found in nearly-permanent social units that include related, as well as unrelated, animals[9]. Mature males lead quasi-solitary lives and spend a great part of their lives near the poles, associate only briefly with female social units for mating[9], and rarely perform coda

vocalizations[10]. Therefore, males would not play any important role in the behavioural learning mechanisms we addressed here, and thus the simulated social units contained only female agents.

ii. Calves experience high natal group philopatry, i.e. they remain in the natal social unit since they are highly dependent on their mothers[9,11]. Thus, agents with 0, 1 and 2 years old do not change social unit membership, i.e., the probability of remaining with the mothers in their natal social unit (i.e. retaining the social unit membership label) was *rem* = 1.

iii. Changes in social unit membership are very rare. Social units are long-term entities that are composed of females and immatures that live and move together for many years and likely their entire lives[9,12]. Thus, adult females have very low probability of migrating to other social units[13]. There is a rough estimate of 6.3% probability of individuals to be involved in social unit merging, splitting or transferring of individuals within a given year[13]. Thus, in our models there was a very low probability, $c = 0.05$, of adult agents migrating to another social unit (i.e., of randomly changing their social unit label) through their lives. In the abovementioned study[13], the individuals who changed social units were relatively young females. Thus, in our models the migration probability was age-dependent, inversely proportional to the agents' age, with youngster agents more prone to change their social unit.

iv. Therefore, with *i*, *ii*, and *iii*, the social units emerge in the simulations as nearly-permanent and nearly-matrilineal, as in the empirical data. Although four other short-term aggregative levels have been described for sperm whales, none of them are considered a social level *per se*[9] either because they are temporary (small temporal scale: clusters, groups) or because they do not involve social interaction among individuals (large spatial scale: aggregations, concentrations). Clusters are ephemeral sets of individuals swimming closely at the water surface for periods of minutes, and groups are sets of units moving together in a coordinate manner for periods of few hours to few days[9]. Concentrations are patches of whales spanning a few hundred kilometers, within which aggregations of individuals within 10-20 kilometers may occur[9]. Therefore, these short-term aggregative levels were not represented in the models.

b. Population parameters

v. Empirical social units were delineated using individuals re-sighted at least three times, with at least two other individuals during at least two 12h-identification periods spaced out by at least 30 days[13]. Off the Galápagos Islands, there were 174 photo-identified individual sperm whales that meet the above criteria and were assigned to 18 known social units[13]. To account for undersampling, the simulated initial population size was more than 5 times larger (N_0 = 1000 individuals) than the empirical number of photo-identified individuals, but still a reasonable number of sperm whales (excluding mature males) to be using the waters off the Galápagos Islands[14].

vi. The mean social unit size off the Galápagos Islands is about 12 members (including sometimes juvenile males)[14,15]. The maximum initial number of simulated social units was a function of the initial population size: $T_{VU} = N_0 / SU$ where T_{VU} is the total number of social units at time step t=1, N_0 is the initial population size, and SU is a random integer from the uniform distribution ϵ[8,14].

vii. Social units rarely split, merge, or experience abrupt membership changes[9,13]. With stochastic demographic processes in the simulations, social units may increase or decrease in size over the simulated time (see Supplementary Fig. 3A). To prevent the simulated social units from growing indefinitely, they split in half when their size doubled the maximum initial social unit size at time step t=1. All agents of a splitting social unit had the same probability of remaining in their original unit or being part of a new social unit. That is, half of the agents of that social unit were randomly selected with equal probabilities to keep their social unit membership labels, while the other half had their social unit membership replaced by the new label T_{vu_t} +1, where T_{vu_t} is the maximum number of social units existent at the time step t.

viii. Although precise ages are hard to estimate empirically, female sperm whales are thought to become sexually mature after at about the age of 9, stop reproducing after about 40 years old, and live 70 years on average[9]. In the simulations, agents followed these characteristics. The simulation were started with agents' age being randomly assigned from an exponential distribution ($f(age) = \lambda \cdot e^{(-\lambda \cdot age)}$, $age \geq 0$, $\lambda = 0.08$), so the initial population was mostly young, with ages typically varying from 0 to 70 years old. Agents reproduced from 9 to 40 years old (i.e. newborn calf agents are added to their simulated social

units), and demographic rates were chosen such that most whales lived 70 years (although some could die earlier and a few live longer) (Supplementary Fig. 3B).

ix. The empirical female sperm whale reproduction rate is age-specific and estimated[16] to be $b_{emp\_i} = 0.257 - (0.0038 \times age_i)$, where b_{emp_i} is the empirical birth rate and age_i is the age in years of the individual i. Because we were interested only in female agents, we simulated female offspring only, so the simulated birth rate was half of the empirical one ($b_i = b_{emp\_i} / 2$), assuming a 1:1 sex ratio in birth events. At each time step t, a birth probability b_{i_t} was calculated for all agents of the population who reached sexual maturity, which have the same probability of giving birth. In the social unit of the selected reproductive agents there will be an addition of one agent with age 0 and an empty coda repertoire vector.

x. Population was modeled as density-dependent. We assumed that all social unit members compete for the same foraged resources, and thus the population fluctuated around the carrying capacity (i.e., the initial population size, N_0 = 1000 individuals) over time (see Supplementary Fig. 3C).

xi. Mortality rates (m_t) were defined as $m_t = [(B_t + N_t) - N_0] / (B_t + N_t)$, where B_t is the number of births in year t, N_t is the population size in year t, and N_0 is the initial population size. Calves usually have higher mortality than adults, and classic and recent empirical estimates corroborate this pattern[9,10]. In our models, to account for age-dependence in mortality we calculated the number of agents that would die in each time step, m_t, and removed agents from the simulation according to empirical estimates of mortality rates[9] (calf = 0.093 $year^{-1}$; adult and juveniles = 0.055 $year^{-1}$). Therefore, all agents in the population at the time step t had a probability of dying according to their age class. The death of selected agents was represented by the removal of their coda repertoires, social unit membership, and age labels from the model.

xii. The simulations lasted for t = 700 years, a period 10 times longer than the empirical life expectancy[9] and long enough to achieve a steady state in the simulations.

c. Learning parameters

xiii. While sperm whales mainly produce echolocation sounds (continuous series of clicks), they also produce codas, stereotypical patterns of 3-40 broadband clicks lasting less than 3 seconds in total[17]. Codas are performed in a social context[9,18,19] almost exclusively by females[10] and their major function seems to be labelling and reinforcing social bonds[20].

xiv. Sperm whale social units have vocal repertoires of about 31 coda types (usually 3-4 commonly used plus about 25 to 28 uncommonly used coda types) [19,21]. In our simulations, agent calves started with coda repertoires of 31 random codas (frequencies of which were drawn from the uniform distribution ϵ[0,100]). Along the simulation, they could change their repertoires and add frequencies of up to 62 coda types, which is twice as long as the empirical coda repertoires to correct for undersampling of empirical data.

xv. Individuals gradually develop their codas in early ages[22,23] and rarely change coda repertoire as adults. The precise age for learning the acoustic repertoire is genuinely difficult to estimate given the impossibilities of experimental intervention with sperm whales in the wild. While the empirical evidence is inherently scarce, it suggests that individuals mainly compose their vocal repertoires at early ages, rather than over the course of their lives. Based on the best available empirical data, we defined a calf as an agent up to 3 years old—since after this age sperm whales are usually larger and begin to wean and perform deeper dives[12]. Learning was modelled such that calf agents change their coda repertoires between the ages of 0 and 3 years old (see Fig. 2, main text).

xvi. To account for copying errors and innovations[6,24], in all models calf agents were subjected to a low probability (0.02) of replacing the frequency of one randomly chosen coda type (62 codas * 0.02 ≈ 1) by a value drawn from the uniform distribution ϵ[0,100] each year (at ages 0, 1 and 2 year old). This effect represented individual learning, i.e. the chance of deliberately creating new coda types (innovation), or randomly creating a new type by copying an existent one with low fidelity (copying error). Therefore, with *xii* and *xiii,* changes in the coda repertoire occurred three times for each calf agent and after the age of 3, their repertoires were fixed.

xvii. Individuals within social units have very similar (but not identical) coda repertoires[21,23,25,26]; and social units of the same clans have similar (but not identical) coda repertoires[21,23]. Therefore, just as with the empirical data[21], we evaluated possible clan segregation based on the coda repertoire of the social units. In our simulations, we used the averaged coda repertoires of the social units to examine whether they cluster in the simulated acoustic networks (i.e., by testing for modularity).

xviii. Social units from different clans in the same area have very distinct coda repertoires[21], which justifies clustering of social units with high coda repertoire similarity as emergent clans in the simulations.

xix. Demographic effects and individual movements—such as migration, immigration and dispersal—can be important drivers of cultural diversity[27]. Our models were explicitly temporally-structured and implicitly spatially-structured. To account for the potential demographic and movement effects in coda transmission, while respecting the structure of the sperm whale societies with nearly-permanent social units, we simulated different levels of population mixing. We replicated all the models with social learning and transmission biases in the three social levels that are relevant for sperm whales: social unit, predefined geographical clans and population. In doing so, it is reasonable to assume that the mean distance between an individual and other members of its unit is less than that between the individual and other members of its clan, which in turn is less than the distance between two members of the whole population. We represented the case in which social units are fairly closed structures by allowing the calf agents to learn only within their own social units. We represented learning between social units that are spatially segregated by starting the simulations with predefined clans mimicking the geographically-segregated clans found in the Atlantic[28], and allowing calf agents to copy from agents of other units, but only from those within their clans. Finally, we represented the case of a completely mixed population by allowing the calf agents learn from any agent in the population.

Differences between empirical and simulated coda types. Codas are stereotypical patterns of 3-40 broadband clicks lasting less than 3 seconds in total[17]. The empirical codas types are classified according

to inter-click intervals, i.e., the proportion of time between the clicks[19,21]. For instance, a coda type labelled as 5R contains 5 regularly-spaced clicks (so 4 equal inter-click intervals), while a 4+1 coda also contains 5 clicks but with an extended period between the 4th and 5th clicks (so 3 equal and one extended inter-click interval). Sperm whales can produce several coda types[19,21]. Here, we simulated the absolute frequency of the different coda types each whale can make, not inter-click intervals that define coda types. In our agent-based models, each agent has a coda repertoire represented by a vector with maximum of 62 elements denoting absolute continuous frequencies of different coda types from 0 (absent) to 100 (always performed coda type). For instance, consider a whale *A* that always performs 5 codas types 3R, 4R, 5R, 4+1, 5+1; and a whale *B* that mostly performs the codas 3R, 4R, 7R but sometimes also performs the codas 6R, 2+1, 3+4, 2+4. The repertoire of a simulated agent representing *A* is given by a vector of 62 elements, 5 of which have frequency 100% and the rest is zeroed. Likewise, the repertoire of the agent *B* is also a 62-element vector but with 3 elements with 100%, 4 elements with intermediate frequency (say 30, 40, 60, 50%) and the rest is zeroed. Therefore, the two vectors differ both in number of zeroed elements and in the frequencies of the non-zeroed ones. Note that we simulated and compared frequencies of use of labelled codas, not the codas themselves, because we are interested in evaluating the diversity of codas individuals end up producing under different coda transmission scenarios (to further quantify and compare the similarity of their coda repertoires).

Differences between empirical and simulated coda transmission. The principal mechanism for the acquisition of coda repertoire is hypothesized to be social learning, in which individuals reproduce the coda types they are exposed to[19,21,23,29,30]. The main goal of our agent-based models is to test which transmission mechanisms, if any, can reproduce the empirical patterns (distinct vocal clans of sperm whales with highly similar coda repertoires), presumably generated by social learning of coda types among whales. Therefore, we modeled changes in the agents' coda repertoires (i.e. changes in the frequency of codas used) occurring due to varieties of social learning and other alternative transmission

processes, to observe under which combination of transmission processes vocal clans (clusters of social units with similar coda repertoire) emerged. Briefly, the transmission processes modelled were: genetic inheritance or vertical transmission (agents copy the exact mother's repertoire), individual learning (agents are assigned to random coda types and frequencies), and oblique social learning (see main text and Fig. 2).

Oblique social learning was simulated as agents copying different proportions of other older agents' repertoires (i.e. coda types and their frequencies of usage). For an example of a typical social learning simulated event, consider the two abovementioned fictional whales *A* and *B*. When the agent *A* "learns" from *B*, it randomly selects a coda type *i* from *B'*s repertoire (62-element coda vector) and copies their frequency of use so the *i*th element in *A*'s repertoire will contain the same value as the *i*th element in *B*'s repertoire. The proportion of coda types copied is predefined, as well as from whom the codas were copied (only from individuals of the same social unit; or from individuals of different social units of the same vocal clan; or from any individuals of the population) (see main text and Fig. 2A, iii). This "pure" social learning could also be biased by homophily, conformism, and/or symbolic marking. In the models with homophily, agents copied coda types from agents of social units with high coda repertoire similarity to the social unit they belong to, but not their own (Fig. 2A, iv). In the models with conformism, agents disproportionately copied the most common coda types (within their social unit, within their clan, or within the entire population) (Fig. 2A, v). Finally, in the models with symbolic marking, agent calves copied a specific subset (~10%) of the coda repertoire of their social unit to mark the social unit they belong to. This subset is represented by 6 coda types that individuals from a given social unit will always perform (i.e., all agents will have 6 specific elements with 100% frequencies in the coda vector) (Fig. 2A, vi). In our 20 sub-models, these transmission processes could be combined and occur within the social unit (Fig. 2B, vii), the pre-defined vocal clan (Fig. 2B, viii), or entire population (Fig. 2B, xi) (see also the main text and Fig. 3A for full description).

Differences between empirical and simulated coda repertoire comparison. Empirical coda repertoires were assigned to the social units whose members were photo-identified within 2h of the recording at the field and had at least 25 codas recorded. The empirical repertoires of the social units were compared using two methods, one based on continuous and other on categorical measures[21,31]. The first method used an averaged multivariate similarity method to compare codas with the same number of clicks, based on the infinity-norm distance between the absolute and standardized intervals between adjacent clicks (inter-click intervals): $s_{AB} = \frac{\sum_{i=1}^{n_A} \sum_{j=1}^{n_B} {}_{l_j = l_i} \frac{0.001}{(0.001 + d_{ij})}}{(n_A \cdot n_B)}$, where s_{AB} is the coda similarity between the repertoires A and B, each with n_A and n_B codas respectively; l_i is the number of clicks in coda i of set A, l_j is the number of clicks in coda j of set B, and d_{ij} is the maximum distance (given by the infinity-norm) between the inter-click interval vectors of the codas i and j. The basal similarity was 0.001 to give a very fine scale comparison of codas on the order of 1 millisecond (although results are robust to variation)[31]. The multivariate similarity (s) was used to build the links among social units in the empirical acoustic networks, which we then used to analyze the clan partition. The second method categorized codas into nearly discrete types based on the number of clicks using a k-means clustering algorithm and a variance ratio criterion to choose the number of clusters[19,21]. Each coda type was given a representative label (e.g. 3R for codas with three regularly spaced clicks; 4+1 for 5-click codas with an extended interval before the last click). Two categorical coda types were considered similar if they were assigned to the same type (categorical similarity = 1) and dissimilar if assigned to different types (categorical similarity = 0) [19,21].

In our agent-based models, we simulated frequencies of usage of coda types (categories), and not the inter-click intervals of each coda, therefore the coda repertoire comparison resemble the second method. We chose the asymmetric weighted Bray-Curtis index as a feasible metric to compare the simulated data—the averaged frequency of usage of codas within members of a social unit—to calculate the similarity between the repertoires of the social units and further evaluate the emergence of clans. For each two coda repertoires, the similarity of the frequencies of use of coda types was given by: $BC_{AB} = 1 - \left(\frac{2C_{AB}}{S_A + S_B}\right)$ where C_{AB} is the sum of the lowest frequency of the coda types that are common to both

repertoires of the social unit *A* and *B*; S_A is the total frequency of the codas in the repertoire of the social unit *A*, while S_B is the total frequency of codas in the repertoire of the social unit B. The original Bray-Curtis index measures dissimilarity, so here $1- BC_{AB}$ gives the similarity between repertoires: from 0 (completely different) to 1 (exactly equal repertoires). Note that this metric did not consider coda types that were simultaneously absent (double zeroes) in both repertoire vectors. Finally, the Bray-Curtis similarities between simulated social units were used to build the simulated acoustic networks from which we analyzed the potential partition into clans.

Sensitivity analysis of parameterization and initial conditions in the agent-based models. Agent-based models usually rely on assumed parameters to modulate the agents' behaviour. In some cases, their empirical values are unknown. We have grounded the parameter estimates of our models using the best empirical evidence available (see Empirical support for the agent-based models, Supplementary Methods). In our main analysis (Figs. 3 and 4, main text), the parameters and initial conditions were fixed across scenarios, so we can directly compare differences in learning strategies—the overarching goal of this study—without having the confounding influence of changing parameter values or starting conditions.

Possibly, different starting points in the simulated scenarios could cascade into different model outputs. To evaluate the robustness of our findings on the partition of simulated social units of sperm whales into clans with distinct acoustic behaviour, we performed a sensitivity analysis of the parameters that were common to all of the 20 agent-based models. We assessed the impact of 6 initial demographic parameters in addition to 2 key parameters of coda transmission (Supplementary Table 2). Some demographic parameters could affect the number of learners (calf agents) and tutors (adult agents) in the population: *population size* (carrying capacity) controls number of agents; *reproductive age* controls addition of agents; *mortality* controls removal of agents; *initial age distribution* controls number of calves in the starting populations. Others tested demographic parameters could affect the probabilities of

interaction between agents: *migration rates among social units* affect population mixing; *average social unit size* affect number of associates of an agent. Finally, the coda transmission parameters could affect learning fidelity and cultural trait diversity: *individual learning* controls the rate of innovations and leaning errors in producing new coda types; while *coda repertoire length* controls number of coda types to be learned.

To evaluate the clan partition, we ran the models changing a single parameter value at a time and calculated the modularity *Q*-metric for weighted one-mode networks, our measure of clan partition. Significantly high *Q*-values indicated a reliable partition in the acoustic network into modules, i.e., the emergent clans. The significance of *Q*-values was checked using an appropriate theoretical model reshuffling the link weights among social units, using 1000 iterations. Modularity was considered high and significant when the observed *Q*-value was higher than the expected by chance, falling outside of the 95% confidence intervals (CI) of the benchmark distribution generated by the theoretical model (for more details, see details in the main methods section).

Our models are computationally demanding; therefore, for each of the 8 parameters and initial conditions we tested two values (the extremes of a biologically-meaningful range) and replicated it 10 times for each model, amassing 3200 *Q*-values from independent runs of the agent-based models. Supplementary Table 2 displays all parameters and their tested ranges. We compared our modularity findings (Figs. 3C and 4, main text) with 16 other situations, changing a single parameter at a time. This allowed us to parse out the effect of each parameter on our modularity results, rather than having confounding influences of multiple parameter changes as a time. While, ideally, we would examine interactions of parameters as well, due to computational limitations with agent-based models, it is common to examine changing a single parameter at a time[32,33]. The results are available in the Supplementary Figure 1 and Supplementary Note 1.

Sensitivity analysis of clan partition. We defined a clan, both in the empirical and simulated datasets, using a weighted modularity metric to evaluate the partition of the acoustic networks (i.e., nodes representing social units connected by weighted links representing similarity among their coda repertoires). Although the agent-based models simulated coda repertoires for each agent, the empirical data is not resolved at the individual level since it is logistically very difficult to assign coda production to individual whales in the wild (this has been achieved in just one case[26]). To make the simulated data comparable to the empirical data, we averaged the coda repertoires of all agents of the social units to build the acoustic networks (see Fig. 4, main text). However, the simulation of coda repertoires with agent-based models represents a "complete sampling", in the sense that we are able to record all codas of all agents of all social units. Clearly, this is not the case for the empirical data, for the same logistical challenges.

We have used the weighted counterpart of the modularity metric to account for fine-scale variation in the similarities among simulated social units. To evaluate if the metric is robust to variation in the levels of sampling in the individual coda repertoires—and make simulated and real-world acoustic networks even more comparable—we performed a sensitivity analysis to the resultant weighted modularity Q-values in the simulations. We resampled (with replacement, 1000 iterations) the simulated network links of all 20 ABMs and evaluated the modularity values with increasing sampling gradient, from 5% to 100% with increment of 5% of the links at a time (bootstrap procedure). The results are available in the Supplementary Figure 2 and Supplementary Note 2.

Supplementary References

1. Chiquet, R. A., Ma, B., Ackleh, A. S., Pal, N. & Sidorovskaia, N. Demographic analysis of sperm whales using matrix population models. *Ecol. Model*. **248**, 71-79 (2013).

2. Whitehead, H. & Gero, S. Positive observed rates of increase do not reflect a healthy population: conflicting rates of increase in the sperm whale population of the eastern Caribbean. *Endanger. Species Res.* **27**, 207-218 (2015).

3. Williams, J. & Slater, P. Modeling bird song dialects—the influence of repertoire size and numbers of neighbours. *J. Theor. Biol.* **145**, 487–496 (1990).

4. Ellers, J. & Slabbekoorn, H. Song divergence and male dispersal among bird populations: a spatially explicit model testing the role of vocal learning. *Anim. Behav.* **65**, 671–681 (2003).

5. Nunn, C., Thrall, P., Bartz, K., Dasgupta, T. & Boesch, C. Do transmission mechanisms or social systems drive cultural dynamics in socially structured populations? *Anim. Behav.* **77**, 1515–1524 (2009).

6. Slater, P. J. The cultural transmission of bird song. *Trends Ecol Evol*. **1**, 94–97 (1986).

7. Whiten, A., Goodall, J., McGrew, W., Nishida, T. & Reynolds V. Charting cultural variation in chimpanzees. *Behaviour* **138**, 1481–1516 (2001).

8. Cantor, M. & Whitehead, H. The interplay between social networks and culture: theoretically and among whales and dolphins. *Phil. Trans. R. Soc. B* **368**, 20120340 (2013).

9. Whitehead, H. *Sperm Whales: Social Evolution in the Ocean*. (Chicago University Press, 2003).

10. Marcoux, M., Whitehead, H. & Rendell, L. Coda vocalizations recorded in breeding areas are almost entirely produced by mature female sperm whales (*Physeter macrocephalus*). *Can. J. Zool.* **84**, 609-614 (2006).

11. Gero, S., Gordon, J. & Whitehead, H. Calves as social hubs: dynamics of the social network within sperm whale units. *Proc. R. Soc. B.* **280,** 20131113 (2013).

12. Gero, S. *et al.* Behavior and social structure of the sperm whales of Dominica, West Indies. *Mar. Mamm. Sci*. **30**, 905-922 (2014).

13. Christal, J., Whitehead, H. & Lettevall, E. Sperm whale social units: variation and change. *Can. J. Zool.* **76,** 1431-1440 (1998).

14. Whitehead, H., Christal, J. & Dufault S. Past and distant whaling and the rapid decline of sperm whales off the Galapagos Islands. *Conserv. Biol.* **11**, 1387-1396 (1997).

15. Whitehead, H., Antunes, R., Gero, S., Wong, S. N. P., Engelhaupt, D., & Rendell, L. Multilevel societies of female sperm whales (*Physeter macrocephalus*) in the Atlantic and Pacific: Why are they so different? *Intl. J. Primatol.* **33,** 1142-1164 (2012).

16. Whitehead, H. Estimates of the current global population size and historical trajectory for sperm whales. *Mar. Ecol. Prog. Ser.* **242,** 295-304 (2002).

17. Watkins, W. A. & Schevill, W. E. Sperm whale codas. *J. Acoust. Soc. Am*. **62**,1486-1490 (1977).

18. Whitehead, H. & Weilgart, L. Patterns of visually observable behavior and vocalizations in groups of female sperm whales. *Behaviour* **118,** 271-296 (1991).

19. Weilgart, L. & Whitehead, H. Group-specific dialects and geographical variation in coda repertoire in South Pacific sperm whales. *Behav. Ecol. Sociobiol.* **40,** 277-285 (1997).

20. Schulz, T. M., Whitehead, H., Gero, S. & Rendell, L. Overlapping and matching of codas in vocal interactions between sperm whales: insights into communication function. *An. Behav.* **76,** 1977-1988 (2008).

21. Rendell, L. & Whitehead, H. Vocal clans in sperm whales (*Physeter macrocephalus*). *Proc. R. Soc. B* **270,** 225-231 (2003).

22. Watkins, W. A., Moore, K. E., Clark, C. W. & Dahlheim, M. E. in *Animal Sonar* (eds Nachtigall, P.E. & Moore, P.W.B.) 99-107 (Plenum, 1988).

23. Gero, S. *On the Dynamics of Social Relationships and Vocal Communication between Individuals and Social Units of Sperm Whales*. Ph.D. Thesis (Dalhousie University, 2012).

24. Filatova, O. A., Burdin, A. M. & Hoyt, E. Is killer whale dialect evolution random? *Behav. Proc.* **99**, 34–41 (2013).

25. Rendell, L. & Whitehead, H. Do sperm whales share coda vocalizations? Insights into coda usage from acoustic size measurement. *An. Behav.* **67,** 865-874 (2004).

26. Schulz, T. M., Whitehead, H., Gero, S. & Rendell, L. Individual vocal production in a sperm whale (*Physeter macrocephalus*) social unit. *Mar. Mam. Sci.* **27,** 149-166 (2010).

27. Fayet, A. L., Tobias, J. A., Hintzen, R. E., & Seddon, N. Immigration and dispersal are key determinants of cultural diversity in a songbird population. *Behav. Ecol*. **25**, 744-753 (2014).

28. Antunes, R. N. C. *Variation in Sperm Whale (*Physeter macrocephalus*) Coda Vocalizations and Social Structure in the North Atlantic Ocean*. PhD thesis. (University of St. Andrews, 2009).

29. Rendell, L., Mesnick, S. L., Dalebout, M. L., Burtenshaw, J. & Whitehead, H. Can genetic differences explain vocal dialect variation in sperm whales, *Physeter macrocephalus*? *Behav. Gen.* **42,** 332-343 (2012).

30. Whitehead, H. & Rendell, L. *Oceans of Culture: Cultures of Whales and Dolphins*. (Chicago University Press, 2014).

31. Rendell, L. & Whitehead, H. Comparing repertoires of sperm whale codas: a multiple methods approach. *Bioacoustics* **14**, 61-81 (2003).

32. Czaczkes, T. J. How to not get stuck—Negative feedback due to crowding maintains flexibility in ant foraging. *J. Theor. Biol.* **360**, 172-180 (2014).

33. Kanagaraj, R., Wiegand, T., Kramer-Schadt, S. & Goyal, S. P. Using individual-based movement models to assess inter-patch connectivity for large carnivores in fragmented landscapes. *Biol. Conserv.* **167**, 298-309 (2013).
